# Combined management can decrease blood pressure: an investigation of health-seeking behaviors among hypertensive patients in urban communities in China

**DOI:** 10.1186/s12872-021-02073-8

**Published:** 2021-05-25

**Authors:** Si Wang, Kai Liu, Xin Zhang, Qingtao Meng, Xinran Li, Runyu Ye, Zhipeng Zhang, Xiaoping Chen

**Affiliations:** Department of Cardiovascular Medicine, West China Hospital, Sichuan University, Chengdu, 610041 China

**Keywords:** Health-seeking behaviours, Combined management, Community health service centre, Blood pressure, Hypertension

## Abstract

**Background:**

Hypertensive patients can freely choose informal medical facilities, such as pharmacies, community health service centres, and cardiology clinics in secondary or tertiary hospitals, as routine places for medical treatment in China currently. The proportions, influencing factors and effects of different health-seeking behaviours on blood pressure (BP) among hypertensive patients in urban communities are not clear. The aim of the study was to investigate health-seeking behaviours and the effects of different health-seeking behaviours on BP among hypertensive patients in urban communities in China.

**Methods:**

A cross-sectional survey of hypertension was conducted in urban communities in Chengdu. A total of 437 hypertensive patients seeking medical help regularly were sequentially enrolled to complete a the questionnaire on health-seeking behaviours.

**Results:**

The average age was 67.1 ± 7.5 years old. The control rate of BP was 41.0%, and the systolic blood pressure (SBP) and diastolic blood pressure (DBP) were 144.2 ± 17.9 mm Hg and 75.4 ± 10.4 mm Hg, respectively. Among the hypertensive patients investigated, 62.8% chose community health service centre, 5.2% chose informal medical facilities, 21.5% chose cardiology clinics in secondary or tertiary hospitals, and 10.5% chose both community health service centre and cardiology clinics as the usual places for medical treatment. There were significant differences in education levels, proportions of home BP monitoring, establishment of chronic disease archives in the community, medication adherence and side effects of drugs among the four groups. The control rates of BP were 39.4%, 23.8%, 43.0% and 54.8% (*P* = 0.100), respectively. The SBPs were 145.1 ± 18.0, 150.9 ± 19.8, 143.8 ± 17.5 and 136.3 ± 15.1 mm Hg (*P* = 0.007), respectively, and it was significantly lower in the combined management group than in the other three groups. Compared with patients choosing community health service centre, patients in the combined management group had a significantly lower BP level (β = −0.119, *P* = 0.038) adjusting for age, sex, education level, establishment of chronic disease archives, medication adherence and number of antihypertensive drugs.

**Conclusions:**

Combined management with both community health service centre and higher-level hospitals can decrease BP.

## Background

Hypertension is a common cardiovascular risk factor that can cause damage to the heart, brain and kidneys and can give rise to the occurrence of cardiovascular events, such as heart failure, stroke and renal failure. Decreasing blood pressure (BP) to the target can greatly reduce the incidence of cardiovascular events in patients with hypertension [1, 2]. Hypertension has a high prevalence in China, occurring in nearly 23.9% of people older than 18 years old [3], but the control rate is very low – only 5.7% in a population-based screening study [4] and 38.3% in urban areas in another cross-sectional study in China [5].

Conducting health management in hypertensive patients relying on the community health service centres is an important part of basic public health services in China. Patients with hypertension were advised to establish chronic disease archives and be followed up in the community health service centres according to the guideline [6]. Standardized hypertension management can significantly improve the hypertension control rate in the community [7–9]. However, due to the different quality of basic health services provided by different communities and the different demands of hypertensive patients, not all hypertensive patients consider community health service centres to be routine places for medical treatment [10–12]. They can freely choose informal medical facilities without physicians in attendance, community health service centres, and cardiology clinics in secondary or tertiary hospitals as routine places for medical treatment. Currently, the proportions, influencing factors and effects of different health-seeking behaviours on BP among hypertensive patients in urban communities in China are not clear.

Therefore, we conducted an investigation of the health-seeking behaviours among hypertensive patients in an urban community in Chengdu, a central city in western China, to determine the health-seeking behaviours of hypertensive patients and the influencing factors and to reveal the effects of different health-seeking behaviours on BP.

## Methods

### Study design

**Fig. 1:**
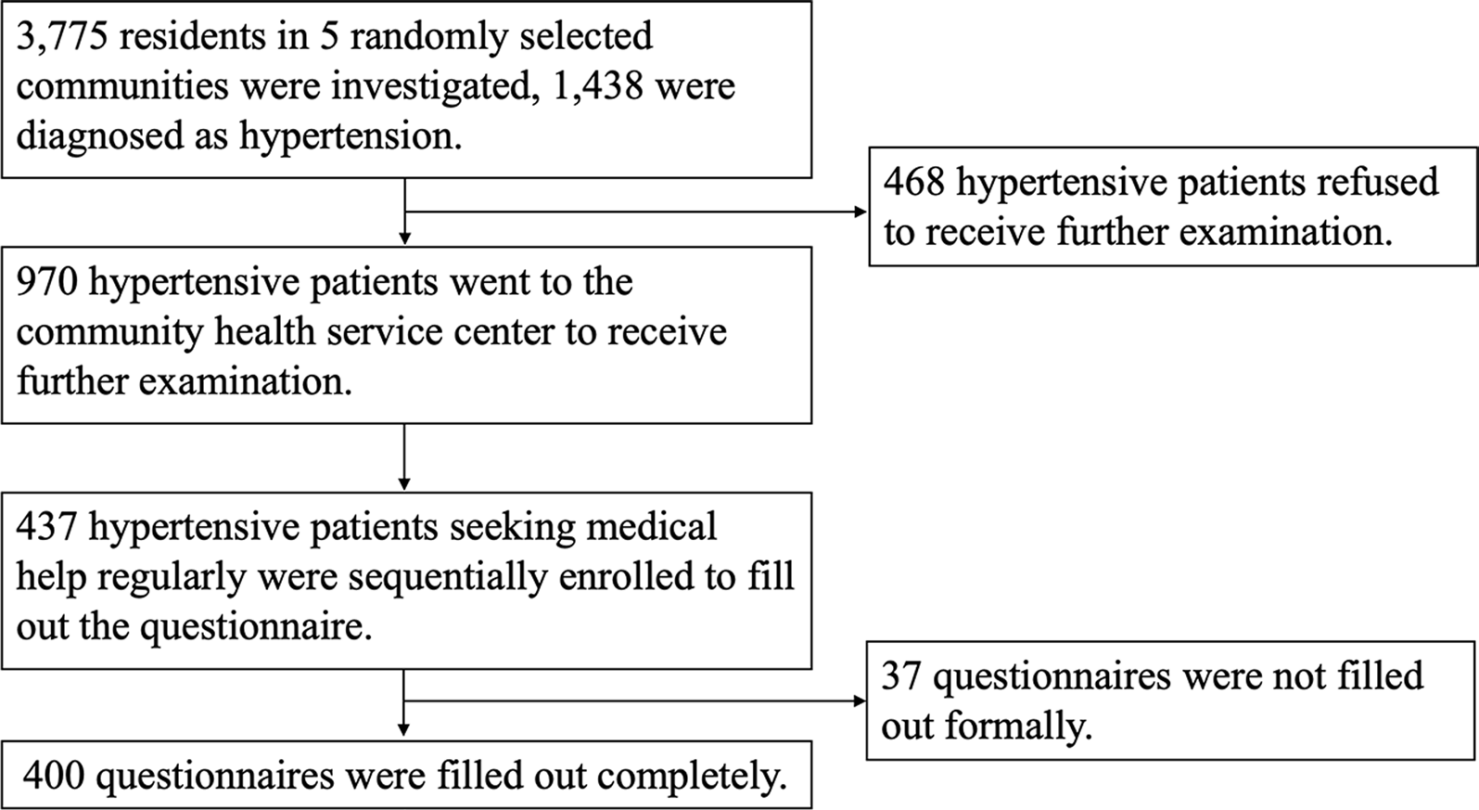
Recruitment of survey participants

We conducted a cross-sectional survey of hypertension in residents older than 18 years old through door-to-door visits and registration in community activity centres using a random cluster sampling method in 5 randomly selected urban residential communities in Chengdu belonging to the same community health service centre in 2011–2012. As shown in Fig. 1, a total of 3,775 residents were investigated, and 1,438 residents were diagnosed with hypertension. A total of 970 hypertensive patients voluntarily visited community health service centre to receive physical examinations and blood tests. A total of 437 hypertensive patients seeking medical help regularly were sequentially enrolled by trained staffs to complete the questionnaire on health-seeking behaviours. Finally, 400 questionnaires were completed in a standardized manner, and the recovery rate was 91.5%. The study protocol was carried out in accordance with the Declaration of Helsinki and was approved by the ethics committee of West China Hospital, Sichuan University. Written informed consent was obtained from all of the participants.

### Measurements

Basic information included name, age, sex, currently smoking, education level, combined cardiovascular diseases, duration of hypertension, performing home BP monitoring, establishment of chronic disease archives, medication adherence, side effects of drugs, antihypertensive drugs and medical insurance reimbursement. Health-seeking behaviours referred to patients' different choices of medical treatment places. For physical examinations, the patients were asked to sit for at least 5 min, and then 3 consecutive BP readings were recorded from the supported right arm at one-minute intervals using validated OMRON HEM-7200 sphygmomanometers (Omron (Dalian) Co., Ltd, China). The BP was calculated as the average value of the three measurements. The other anthropometric data, including height, weight and waist circumference, were measured as before [13]. Blood tests including creatinine, uric acid (UA), glucose, triglyceride (TG), total cholesterol (TC), high-density lipoprotein cholesterol (HDL-C) and low-density lipoprotein cholesterol (LDL-C) were measured using a Hitachi 7600 analyzer (Hitachi High-Technologies, Tokyo, Japan).

### Related definitions

Hypertension was defined as the calculated average systolic blood pressure (SBP) ≥ 140 mm Hg and/or diastolic blood pressure (DBP) ≥ 90 mm Hg or taking antihypertensive drugs [14]. Diabetes was defined as fasting plasma glucose ≥ 7.0 mmol/L or taking hypoglycaemic drugs [15]. Body mass index (BMI) was calculated as body mass/height^2^ (kg/m^2^). Obesity was defined as BMI ≥ 28 kg/m^2^ or waist circumference ≥ 90 cm for men and ≥ 85 cm for women [16, 17]. Smoking was defined as smoking more than 1 cigarette per day. BP control was defined as BP controlled at less than 140/90 mm Hg. Combined cardiovascular diseases indicated patients were diagnosed as atrial fibrillation, coronary heart disease, heart failure or stroke in the hospital before. Performing home BP monitoring indicated patients would like to measure their BPs at home by sphygmomanometers sometimes. Good medication adherence indicated taking antihypertensive medications for more than 9 months per year through the questionnaires.

### Statistical analysis

Patients with hypertension were divided into four groups according to the different places that they chose for medical treatment. Group 1 consisted of patients choosing community health service centre as the usual place for medical treatment; group 2 consisted of patients choosing informal medical facilities without physicians in attendance, such as pharmacies, as the usual places for medical treatment; group 3 consisted of patients choosing the cardiology clinics in secondary or tertiary hospitals as the usual places for medical treatment; and group 4, named the combined management group, consisted of patients choosing both community health service centre and higher-level hospitals as the usual places for medical treatment. Descriptive data are presented as the means (SD, standard deviation) and proportions. The F test was used for comparison of the characteristics among the 4 groups, and the LSD test was used for pairwise comparisons between groups. Comparison of categorical variables among 4 groups was evaluated with the Pearson’s χ2 test. The influences of different health-seeking behaviours on the BP control rate and BP level were further analysed using logistic regression analysis and linear regression analysis respectively by taking different groups as categorical variables and group 1 as a reference. The adjusted model was conducted adjusted for age, sex, education level, establishment of chronic disease archives, medication adherence and number of antihypertensive drugs. The SPSS software package (IBM Corp. Released 2019. IBM SPSS Statistics for Windows, version 26.0. Armonk, NY, USA: IBM Corp) was used to conduct the statistical analysis. Statistical significance was defined as P < 0.05.

## Results

### Basic information of the hypertensive patients

The average age of the hypertensive patients investigated was 67.1 ± 7.5 years old, 46.5% of whom were male. The average duration of hypertension was 10.1 ± 8.7 years. The total control rate of blood pressure was 41.0%, and the SBP and DBP were 144.2 ± 17.9 mmHg and 75.4 ± 10.4 mm Hg, respectively. Among the participants, 18.3% had a cultural degree of primary school or less, 43.2% had a junior high school education, and 38.5% had a high school level of education or more, and the proportion of smoking was 9.0%. A total of 43.4% of the participants were diagnosed with obesity and 22.0% were diagnosed as having diabetes. 66.3% performed home BP monitoring, 75.5% established chronic disease archives in community health service centre, 85.0% had good medication adherence, 22.5% had side effects of antihypertensive drugs, and 60.8% could receive partial reimbursement.

### Investigation of patients' health-seeking behaviours and the influencing factors

The proportions of different health-seeking behaviours were shown in Table 1. There were no significant differences in age, sex, waist circumference, BMI, duration of hypertension, the results of blood tests or proportions of currently smoking, obesity, diabetes, combined cardiovascular diseases, medical insurance reimbursement or antihypertensive drugs among groups with different health-seeking behaviours. Otherwise, there were significant differences in the proportions of education levels, home BP monitoring, establishment of chronic disease archives in the community, medication adherence and side effects of drugs among the four groups.

**Table 1:** The characteristics of the participants in the four groups

Characteristics	Group 1	Group 2	Group 3	Group 4	P value
Number	251 (62.8)	21 (5.2)	86 (21.5)	42 (10.5)	
Age	67.0 ± 7.3	67.9 ± 7.7	67.9 ± 7.1	65.3 ± 9.1	0.283
Sex (male)	113 (45.0)	8 (38.1)	47 (54.7)	18 (42.9)	0.346
Waist, cm	85.3 ± 8.6	85.4 ± 8.2	86.2 ± 9.0	87.6 ± 10.4	0.474
BMI, kg/cm^2^	24.5 ± 2.8	25.1 ± 4.1	24.5 ± 3.2	24.7 ± 2.9	0.831
Currently smoking	22 (8.8)	2 (9.5)	9 (10.5)	3 (7.1)	0.917
*Education levels*					
Primary school or less	51 (20.3)	8 (38.1)	7 (8.1)	7 (16.7)	0.028
Junior high school	107 (42.6)	9 (42.9)	40 (46.5)	17 (40.5)	
High school or more	93 (37.1)	4 (19.0)	39 (45.3)	18 (42.9)	
Obesity	108 (43.0)	8 (38.1)	34 (39.5)	23 (54.8)	0.394
Diabetes	51 (20.3)	4 (19.0)	23 (26.7)	10 (23.8)	0.64
Combined cardiovascular diseases	55 (21.9)	4 (19.0)	22 (25.6)	8 (19.0)	0.845
Duration of hypertension	9.6 ± 8.8	10.1 ± 7.1	11.2 ± 8.7	10.5 ± 8.7	0.544
Home BP monitoring	149 (59.4)	16 (76.2)	67 (77.9)	33 (78.6)	< 0.001
Chronic disease archives	202 (80.5)	11 (52.4)	53 (61.6)	36 (85.7)	< 0.001
Medication adherence	207 (82.1)	17 (81.0)	75 (87.2)	41 (97.6)	0.033
Side effects of drugs	61 (24.3)	4 (19.0)	11 (12.8)	14 (43.8)	0.005
*Antihypertensive drugs*					
1 drug	176 (70.1)	15 (71.4)	51 (59.3)	27 (64.3)	0.220
2 drugs	61 (24.3)	6 (28.6)	31 (36.0)	10 (23.8)	
≥ 3 drugs	14 (5.6)	0 (0)	4 (4.7)	5 (11.9)	
Medical insurance reimbursement	152 (60.6)	9 (42.9)	55 (64.0)	27 (64.3)	0.338
Creatinine, μmol/L	91.9 ± 15.2	94.9 ± 18.7	96.2 ± 16.9	92.4 ± 14.4	0.323
UA, μmmol/L	363.1 ± 94.8	363.7 ± 111.0	395.4 ± 95.2	340.6 ± 91.8	0.066
Glucose, mmol/L	5.94 ± 1.56	6.44 ± 2.41	6.39 ± 1.67	5.97 ± 1.25	0.227
TG, mmol/L	1.60 ± 0.72	1.70 ± 0.69	1.77 ± 1.01	1.60 ± 0.93	0.543
TC, mmol/L	5.20 ± 3.03	5.28 ± 0.87	4.85 ± 1.04	4.96 ± 1.08	0.783
HDL-C, mmol/L	1.48 ± 0.33	1.65 ± 0.51	1.41 ± 0.34	1.52 ± 0.33	0.076
LDL-C, mmol/L	2.50 ± 0.71	2.66 ± 0.92	2.56 ± 0.72	2.42 ± 0.79	0.823

Data are presented as means ± SD or number (percentage)

BMI: body mass index; UA: uric acid; TG: triglyceride; TC: total cholesterol; HDL-C: high density lipoprotein-cholesterol; LDL-C: low density lipoprotein-cholesterol

### Effects of different health-seeking behaviours on BP control rate

**Fig. 2:**
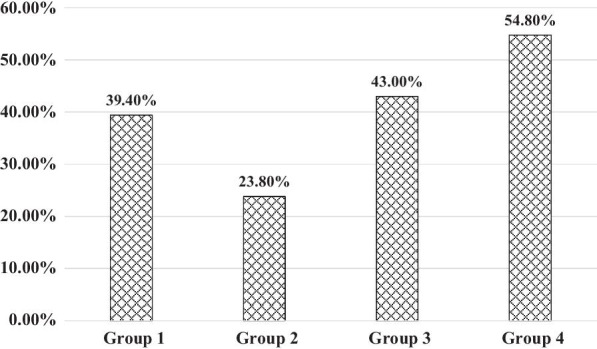
The control rates of BP in the four groups

The control rates of BP in the four groups were shown in Fig. 2, there was no significant difference among the four groups (*P* = 0.100). Table 2 showed the results of logistic regression analysis for the relationship of health-seeking behaviours and BP control. Compared with patients in group 1, who chose community health service centre as the usual place for medical treatment, patients in the combined management group didn’t have a significantly higher BP control rate in the unadjusted model and adjusted model after adjusting for age, sex, education level, establishment of chronic disease archives, medication adherence and number of anti-hypertensive drugs.

**Table 2:** Logistic regression analysis for the influence of health-seeking behaviors on BP control

	Unadjusted			Adjusted		
	OR	95% CI	P value	OR	95% CI	P value
*Reference* = *Group 1*						
Group 2	0.480	0.17–1.351	0.165	0.429	0.129–1.427	0.167
Group 3	1.159	0.706–1.904	0.559	1.243	0.678–2.279	0.482
Group 4	1.859	0.962–3.59	0.065	1.575	0.742–3.344	0.236

The adjusted model was conducted adjusting for age, sex, education level, establishment of chronic disease archives, medication adherence and number of anti-hypertensive drugs

OR, odds ratio; CI, confidence interval

### Effects of different health-seeking behaviours on BP level

**Fig. 3:**
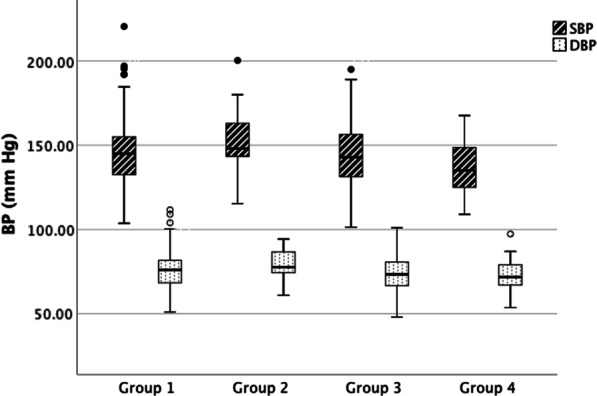
The BPs in the four groups. BP: blood pressure**;** SBP: systolic blood pressure; DBP: diastolic blood pressure. *P < 0.05 when compared with SBP in group 4 through pairwise comparison

As shown in Fig. 3, the SBPs in the four groups were 145.1 ± 18.0, 150.9 ± 19.8, 143.8 ± 17.5 and 136.3 ± 15.1 mm Hg, respectively, and the difference was statistically significant (*P* = 0.007). The SBP in the combined management group was significantly lower than that in the other three groups through pairwise comparison. The DBPs in the four groups were 75.9 ± 10.5, 79.0 ± 8.5, 74.3 ± 10.7 and 72.6 ± 9.7 mm Hg, respectively, and the difference was not statistically significant (*P* = 0.066). Table 3 showed the relationship of health-seeking behaviours and BP level through linear regression analysis. Compared with patients in group 1, patients in the combined management group had a significantly lower BP both in the unadjusted model (β = −0.151, *P* = 0.003) and adjusted model (β = −0.119, *P* = 0.038) after adjusting for age, sex, education level, establishment of chronic disease archives, medication adherence and number of anti-hypertensive drugs.

**Table 3:** Linear regression analysis for the influence of health-seeking behaviors on BP level

	Unadjusted			Adjusted		
	β	95% CI	P value	β	95% CI	P value
*Reference* = *Group 1*						
Group 2	0.073	− 2.059 to 13.744	0.147	0.078	− 2.684 to 14.845	0.173
Group 3	− 0.029	− 5.603 to 3.09	0.57	-0.055	− 7.397 to 2.651	0.353
Group 4	− 0.151	− 14.619 to − 3.021	0.003	-0.119	− 12.954 to -0.355	0.038

The adjusted model was conducted adjusting for age, sex, education level, establishment of chronic disease archives, medication adherence and number of anti-hypertensive drugs

CI, confidence interval

## Discussion

Currently, few studies have reported the medical institution choices of hypertensive patients. Dafeng et al. investigated the health-seeking behaviours of 510 patients with hypertension or diabetes in Wuhan, China, and the results showed that 75.35% of patients chose higher-level hospitals for medical treatment [18]. Another community-based study conducted in Guangdong Province in China including 154,504 subjects who had a usual source of health care, demonstrated that 65.3% of patients used outpatient services at primary care regularly for treating chronic diseases [12]. However, combined health-seeking behaviours were not been reported in the two studies and there were some differences with the results of our study. The population in our study consisted entirely of residents in the central urban area of Chengdu, a central city in western China, with good economic development and community health service construction. We can see that the patients' health-seeking behaviours were completely different due to unbalanced economic development and the different maturity levels of community health service centres in China. Further research must be performed in different regions at different levels of economic development. The results of our study can only partly represent the health-seeking behaviours of hypertensive patients in the central urban areas.

Health-seeking behaviours are defined as any activity undertaken by individuals who perceive themselves to have a health problem or to be ill for the purpose of finding an appropriate remedy [19]. The desired health-seeking behaviour is responding to an illness by seeking help from a trained allopathic doctor at a recognized health care centre [20]. Musinguzi GS et al. found that factors influencing health-seeking behaviours were related to health systems and the patients’ socioeconomic and structural environment [19]. The main system issues were related to availability and attitudes of staff and shortages of supplies and medicines [21]. The patients’ factors were related to awareness, perceived severity, perceived effectiveness of therapy, adverse effects, and perceived fears of lifelong dependence on medicines [22]. However, the factors influencing different health-seeking behaviours are not clear currently. Neither of the two studies that we mentioned before conducted further research on the factors influencing the different health-seeking behaviours [12, 18]. Our studies demonstrated that education levels and the side effects of drugs were related to the patients' health-seeking behaviours. The patients choosing higher-level hospitals as the usual places for medical treatment had the largest proportion of middle and advanced education levels, and the patients choosing the informal medical facilities as the usual places for medical treatment had the largest proportion of a low education level. This finding is just the same as what Fjaer EL et al. found: higher socioeconomic position groups are more likely to use health care specialists than lower socioeconomic position groups, and in the context of health care specialist use, education and occupation appear to be particularly important factors [23]. However, the proportion of patients with middle and advanced educations was not the largest in the combined health-seeking group. This result indicated that education level is not an important factor influencing the choice of combined health-seeking behaviours. Otherwise, the incidence of adverse effects was the highest in the combined group. Studies have shown that adverse effects are an influential factor for drug adherence [24–26]. However, the side effects of drugs could also prompt patients to seek medical treatment, from community health service centre to higher-level hospitals in our study, to receive combined therapy from community health service centre and higher-level hospitals. This behaviour might also occur because patients in the combined management group pay more attention to the drug response.

A community-based epidemiological survey of hypertension showed that the overall control rate of hypertension was poor, as we mentioned before, at only 5.7% in a population-based screening study [4], as well as 38.3% in urban areas and 17.5% in rural areas in another cross-sectional study in China [5]. In a survey conducted in outpatients, including 5206 hypertensive patients from 46 hypertension outpatient clinics in 22 provinces, autonomous regions, and municipalities of China, the control rate of hypertension was 44.3% – higher than the control rate obtained in the community [27]. Our study showed that the BP control rate of hypertensive patients who chose higher-level hospitals as the usual places for medical treatment was 43.0%, similar to the control rate in previous studies [27]. The control rate of hypertensive patients who chose community health service centre as the usual place for medical treatment was 39.4%, which was close to the control rate in the group choosing higher-level hospitals. In addition, patients with hypertension who chose informal medical facilities as the usual places for medical treatment had the lowest BP control rate, which was only 23.8%. The control rate was highest in the combined management group, reaching 54.8%, higher than that in other studies [4, 5, 27], and the average SBP was 136.3/72.6 mm Hg in this group. Why was the level of BP the lowest in the combined management group? There was no significant difference in the number of antihypertensive drugs among the four groups. The proportion of combined medication use was relatively higher in the group choosing the higher-level hospitals for medical treatment, but the BP was lower than that in the group with combined health-seeking behaviours. Therefore, it was not the number of drugs that caused the difference in BP. The rationality of the prescription and the timeliness of prescription adjustment could play more important roles. Drug regimens cannot be more precisely adjusted in patients treated in community health service centres when BP is difficult to control, and medical problems sometimes cannot be solved in a timely manner in patients who simply choose higher-level hospitals for medical treatment. In the combined management group, patients could not only receive medical services from the community health service centre conveniently but also enjoy better medical services from higher-level hospitals. They would like to visit higher-level hospitals if their BP cannot be controlled to normal in the community health service centre and they had the highest medication adherence. However, the loose collaborative management in our study did not significantly improve the BP control rate of hypertensive patients. Yi Q, et al. randomly chose 218 primary hypertensive patients from a community health service centre to investigate the effect of the integration pattern of the hospital-community on the grade-based management of hypertension in elderly individuals. After 6 months of intervention, the control rate in the patients increased from 22.9% to 88.1% [28], which was higher than the BP control rate of the combined management group in our study. However, there was no control group, and the evaluation of health economics and the specific working mode should be further discussed.

Our study has some limitations. First, the sample size was not sufficiently large to obtain the statistically significant difference of BP control rate among groups, but the significant difference of BP among groups suggested that we can expand the sample size to further clarify the relationship. Second, although we performed the survey using a random cluster sampling method, one-third of the hypertensive patients didn’t go voluntarily to the community health service centre to receive physical examinations and blood tests. We need to recognize the selection bias and avoid generalization when interpreting the results. Third, this study was a cross-sectional community sampling survey for evaluating the influence of different health-seeking behaviours on BP. Since the BP was also affected by many other factors that could not be corrected one by one, it could only provide a reference for studies in the future. It is important to further study the effect of hospital-community combined hypertension management on the BP and to further explore reasonable combined management modes. We have performing a study to evaluate the clinical efficacy and health economics effects of an internet-based patient-primary care physician-cardiologist integrated management model of hypertension in China [29]. We look forward to the results.

## Conclusions

In conclusion, more than half of hypertensive patients we surveyed would like to choose community health service centre while ten percent would like to choose both community health service centre and higher-level hospitals as the usual places for medical treatment in the urban communities in China. The levels of education and the side effects of drugs were related to the patients' health-seeking behaviours. Compared with patients choosing community health service centre, patients in the combined management group had a significantly lower BP after adjusting for age, sex, education level, establishment of chronic disease archives, medication adherence and number of anti-hypertensive drugs. However, how to conduct combined management efficiently requires further study.

## Data Availability

The data can be obtained from the corresponding author under reasonable request.

## Abbreviations

BP: Blood pressure
SBP: Systolic blood pressure
DBP: Diastolic blood pressure
UA: Uric acid
TG: Triglyceride
TC: Total cholesterol
HDL-C: High-density lipoprotein cholesterol
LDL-C: Low-density lipoprotein cholesterol
BMI: Body mass index
OR: Odds ratio
CI: Confidence interval
SD: Standard deviation
